# Supplementary material for: What support do frontline workers want? A qualitative study of health and social care workers’ experiences and views of psychosocial support during the COVID-19 pandemic
Source: PLoS One. 2021 Sep 2;16(9):e0256454. doi: 10.1371/journal.pone.0256454 (PMC8412294; doi:10.1371/journal.pone.0256454)
Supplement: S1 Data — This is the interview schedule which was used to guide the semi-structured interviews. (DOCX) [file pone.0256454.s001.docx]

**S1 Data – Frontline Healthcare Workers**

Thank you for agreeing to take part in this interview. The interview should last for about 45 minutes. You can take a break at any time or if you would like to postpone the interview, please do just let me know. If at any point you want to stop the interview and no longer take part, then that is fine too, please just say. Please try to avoid mentioning any identifying features of your colleagues or place of work. If you do, don’t worry, we will just make sure to remove this from the interview transcript. This interview is completely confidential and anonymous, no information about you, your colleagues or your place of work will be identifiable. Are you happy to continue? Thank you.

1. Can you tell me a bit about your usual role?
   - *Where do you usually work?*
   - *What do you usually do in your day to day work?*
2. Tell me about your current role and what work you have been doing in the response to COVID-19?
   - *What is similar/different to your usual role?*
   - *How did this role come about? (i.e. usual role, redeployment, volunteered)*
   - *If redeployed, what was the process of redeployment like for you?*
3. What has been your experience of working on the frontline in response to COVID-19 so far?
   - *How has this changed over time? (explore early, peak, current phases)*
   - *What has your organisation done to support you and your team?*
   - *What measures has your organisation put in place? Have these been helpful?*
4. Can you tell me about how you have felt when doing this work?
5. Have you been faced with any difficult ethical or moral dilemmas in doing this work? Have you had to make any difficult decisions yourself? Or witnessed things which have caused dilemmas for you?
   - *What was the nature of this?*
   - *How did this impact on you?*
   - *How did you manage this?*
6. How, if at all, do you think this work has impacted on your own health and wellbeing?
   - *How has this changed this changed over time? (explore early, peak, current phases)*
   - *Physical and mental health?*
7. What has helped you to cope with this work?
8. What has got in the way of you coping with this work?
9. Have you been offered any psychological support through work so far?
   - *If so, what you have been offered?*
   - *Did you take this up?*
   - *Why did you/did you not take this up?*
10. Have there been any other psychological support options, either made available to you or that you have heard of being offered to others?
    - *What do you think of these other interventions?*
11. Are there forms of support that you would like to have been offered but haven’t?
    - *If so, what and when?*
12. Is there anything which might get in the way of you accessing psychological support even if you had the sense that you might benefit from it?
13. Has the pandemic affected you in your personal life as well?
    - *If so, how has this interacted with you doing your work?*
14. What are your thoughts about how the media has represented healthcare workers during the pandemic?
15. Has there been anything unexpected/that has surprised you in doing this work?
    - *Unexpected gains?*
16. What support do you think you (and your team) might need going forward?
17. If you were put in charge of providing psychological support for healthcare staff like you, what would you recommend?
    - *What types of support?*
    - *Timing of support?*
    - *Delivery of support – i.e. by whom, where?*
18. Is there anything else you would like to tell us?

Thank you for your time. Do you have any questions?
