# Supplementary material for: What support do frontline workers want? A qualitative study of health and social care workers’ experiences and views of psychosocial support during the COVID-19 pandemic
Source: PLoS One. 2021 Sep 2;16(9):e0256454. doi: 10.1371/journal.pone.0256454 (PMC8412294; doi:10.1371/journal.pone.0256454)
Supplement: Attachment — Submitted filename: Response to Reviewers - PLOS One.docx [file pone.0256454.s002.docx]

Dr Jo Billings

UCL Division of Psychiatry

6^th^ Floor, Maple House

149 Tottenham Court Road

London W1T 7NF

Vincenzo De Luca
Academic Editor
PLOS ONE

10^th^ July, 2021

Dear Dr De Luca

**LONDON’S GLOBAL UNIVERSITY**

Thank you to you and your reviewers for your feedback on our manuscript **What support do frontline workers want? A qualitative study of health and social care workers’ experiences and views of psychosocial support during the COVID-19 pandemic (PONE-D-21-07944)**.

We have provided a point-by-point response to yours and the reviewers’ comments in the table below.

| Comments from Academic Editor | | Page |
| --- | --- | --- |
| 1. Please ensure that your manuscript meets PLOS ONE's style requirements, including those for file naming. | We have carefully checked PLOS ONE’s style requirements and amended heading font sizes etc to make sure that the manuscript meets these requirements throughout.  We have renamed the Supplementary File to meet these requirements | -  Supplementary materials |
| 2. In your Methods section, please clarify whether pseudonyms were used. | Pseudonyms were used and we have added this information into the Method section. | Method, p6 |
| 3. We note that you have indicated that data from this study are available upon request. PLOS only allows data to be available upon request if there are legal or ethical restrictions on sharing data publicly. For information on unacceptable data access restrictions, please see http://journals.plos.org/plosone/s/data-availability#loc-unacceptable-data-access-restrictions. | We have clarified this in the Data Availability section. | Declarations, p38 |
| 4. Thank you for stating the following in the Acknowledgments Section of your manuscript:  "This research received no specific grant from any funding agency, commercial or not-for profit sectors. Dr Bloomfield is funded by a UCL Excellence Fellowship and supported by the National Institute for Health Research University College London Hospitals Biomedical Research Centre."  We note that you have provided funding information that is not currently declared in your Funding Statement. However, funding information should not appear in the Acknowledgments section or other areas of your manuscript. We will only publish funding information present in the Funding Statement section of the online submission form.  Please remove any funding-related text from the manuscript and let us know how you would like to update your Funding Statement. Currently, your Funding Statement reads as follows:   "The authors received no specific funding for this work."  Please include your amended statements within your cover letter; we will change the online submission form on your behalf. | Apologies for the confusion. We have deleted the section about Dr Bloomfield’s funding for his fellowship from the manuscript as it is not relevant to this paper.  The funding statement “The authors received no specific funding for this work” is correct.  We are grateful for you updating the online submission form with this information on our behalf. | - |
| 5. Please include captions for your Supporting Information files at the end of your manuscript, and update any in-text citations to match accordingly. | We have included a section on Supporting Information at the end of the manuscript.  We have renamed the file appropriately and uploaded to the editorial manager system.  We have updated the in-text reference to the Supplementary Material. | Supporting Information p37  Supplementary Material  Method, p6 |
| 6. Your ethics statement should only appear in the Methods section of your manuscript. If your ethics statement is written in any section besides the Methods, please delete it from any other section. | In the main manuscript, the ethics statement only appears in the Method section.  We have deleted information about Ethics from the Declarations section. | Method, p6  Declarations, p38 |
| Comments from Reviewer 1 | | |
| Overall well written and informative manuscript. | Thank you for this positive feedback. |  |
| Introduction: Mentioning stats about mental health burden among UK based health professionals may be added. | We have added in an additional reference on a UK healthcare sample and included some key statistics about mental health burden on UK health and social care staff. | Introduction, p4 |
| Methods: There is no clear description about how sample size was achieved. | Please see response to Reviewer 2, below, which also addresses this point. We have added in further information in the Analysis sub-section of the Method section to fully explain our considerations about sample size. | Method – Analysis, p7 |
| Discussion: Mental health awareness and peer support components are two different domains so these should be separately written. Familial support discussion may be added to peer support domain. | We have separated out sections on ‘Peer and family support’ and ‘Mental health awareness’.  We have added in some points about familial support in the section on ‘Peer and family support’. | Discussion, p28-29  Discussion – Peer Support, p28 |
| Add more evidence into discussion part. | We have taken the opportunity to add in further literature which has been published since this manuscript was originally submitted. | Discussion, p28-34. |
| Comments from Reviewer 2 | | |
| The paper is well written, theoretically informed and addresses a subject that had not yet fully covered. This study contributes meaningful and relevant knowledge not only to health and social care workers but also to the entire society about the transverse impact of a pandemic. | Thank you for this very positive feedback, we hope this paper will have a significant impact on health and social care workers, and also the wider systems which support them. | - |
| 1. The sample description requires more detail (in the manuscript and table 1). For example, ages, job or position, outbreak roles, etc. | We have included information on professional role and place of work in the participant information table. We did not gather information about participants’ ages and ethnicities, which is already acknowledged in the Limitations section. The roles that people undertook during the pandemic were multifarious and therefore have not been categorised quantitatively in this study. We have added a line in the limitation section to acknowledge this. | Limitations, p34 |
| 2. Inclusion/exclusion criteria must be specified as well as data saturation criteria, and also a description of participants who were approached but refused to participate in the study. | Inclusion and exclusion criteria have been made clearer in the Methods sections.    The concept of ‘data saturation’ is not considered consistent with the values and assumptions of Reflexive Thematic Analysis, rather it is associated with the constant comparison method of Grounded Theory (please see Braun & Clarke, 2021).  Instead, we drew on the Information Power approach of Malterud, Siersma and Guassora (2016) and sought a pragmatic approach to sample size, as also advocated by Braun and Clarke (2021).  We have added in a line in the Analysis sub-section of the Methods section to fully explain our approach to sample size.  Information about the study was disseminated via social media and by word of mouth through health and social care contacts and potential participants were invited to contact us to take part in the study. There is no way of knowing how many people heard about the study and it is therefore not possible to say what proportion agreed, or refused, to take part from this methodological approach. | Methods – Study design, participants and procedures, p5-6  Methods – Analysis, p7 |
| 3. Regarding the results, themes/sub-themes need to be revisited (they are mostly descriptive). As a qualitative study, a higher analytical depth is expected. | We have tried to present the results of this study in an inductive manner, consistent with the epistemology of Reflexive Thematic Analysis and in line with our stated research aim of “asking frontline health and social care workers about their own experiences and views about psychosocial support during the pandemic, and in so doing, aimed to amplify the voices and views of frontline workers themselves” (p5).  We sought to maintain curiosity about workers’ experiences and views and our results are therefore deliberately presented in a quite descriptive way. We specifically did not seek to transpose any pre-conceived analytical framework or theories on our data.  The results of Thematic Analyses are typically presented in a quite descriptive way, which differs from Grounded Theory and IPA approaches, where the results and discussion sections are typically combined to provide an analytical overview alongside the presentation of results. In this paper, and in line with recommendations for presenting Thematic Analysis (i.e. Braun and Clarke, 2020; Nowell at al., 2017), we have separated out the Results and Discussion sections, and in the Discussion aim to provide this more in-depth analytical narrative, whilst maintaining transparency, trustworthiness and credibility for the reader in how we have interpreted our data.  We have nevertheless taken this feedback on board and have added in some additional comments to ensure that the authors’ voices are maintained throughout the presentation of the Results. | Results, p9-27. |

Thank you again for considering our paper. We hope these amendments address the points raised and very much look forward to hearing further from you.

With kind regards

**Dr Jo Billings**

**Consultant Clinical Psychologist**

**Associate Clinical Professor, Division of Psychiatry, UCL.**


**References**

Braun & Clarke (2021) To saturate or not to saturate? Questioning data saturation as a useful concept for thematic analysis and sample-size rationales. Qualitative Research in Sport, Exercise and Health, 13, 2, 201-216. doi:10.1080/2159676X.2019.1704846

Braun V, Clarke V. One size fits all? What counts as quality practice in (reflexive) thematic analysis? Qual Res Psychol 2020; 14: 1-25.

Malterud, Siersma & Guassora (2016). Sample Size in Qualitative Interview Studies: Guided by Information Power. Qualitative Health Research. 26, 13, 1753-1760. doi:10.1177/1049732315617444

Nowell LS, Norris JM, White DE, Moules NJ. Thematic analysis: Striving to meet the trustworthiness criteria. Int J Qual Methods 2017; 16(1):1609406917733847.
